# Ladybird (Coleoptera: Coccinellidae) Community Dynamics and Climatic Driving Factors in the Taklimakan Desert–Oasis Ecotone

**DOI:** 10.3390/insects17101051

**Published:** 2026-10-13

**Authors:** Aerziguli Rouzi, Wenchao Guo, Qingxuan Xu, Guoyue Yu, Kaiyun Fu, Xiaowu Wang, Xinhua Ding, Tursun Ahmat

**Affiliations:** 1 Key Laboratory of Integrated Pest Management on Crops in Northwestern Oasis, Ministry of Agriculture and Rural Affairs, Urumqi 830091, China; aerzi-insect@foxmail.com (A.R.); gwc1966@163.com (W.G.); fukaiyun000@foxmail.com (K.F.); wxw303528@163.com (X.W.); 2 Institute of Plant Protection, Xinjiang Uygur Autonomous Region Academy of Agricultural Sciences, Key Laboratory of Agricultural Biosafety in Xinjiang, Urumqi 830091, China; 3 Key Laboratory of Crop Stress Biology for Arid Areas, Northwest A&F University, Yangling 712100, China; 4 Institute of Plant Protection, Beijing Academy of Agriculture and Forestry Sciences, Beijing 100097, China; xuqxfarmer@126.com (Q.X.); yuguoyue@aliyun.com (G.Y.)

**Keywords:** ladybird resources, arid agroecosystem, community diversity, desert–oasis ecotone, dominant species

## Abstract

Ladybirds serve as crucial predators in agricultural ecosystems; however, their community dynamics in extremely arid regions are not well understood. We conducted a systematic survey of ladybirds across four habitat types—croplands, orchards, shelterbelts, and weedy margin—at 654 locations in the Taklamakan Desert–oasis ecotone in southern Xinjiang from May to September in 2018, 2019, and 2024. This was done using sweep-netting along with morphological and molecular identification methods. We collected a total of 5414 specimens, representing 23 different species. *Hippodamia variegata* and *Stethorus punctillum* were the most prevalent, making up 80.35% of the total individuals. The highest abundance and α-diversity were observed in June, which is significantly earlier than in temperate regions. Climatic factors alone accounted for the largest unique fraction of community variation (14.2%), followed by the shared habitat–month fraction (13.0%), with all predictor sets together explaining 32.5% of the variation. In this extremely arid region, climate accounted for the largest unique fraction among the measured predictor sets, while habitat and month jointly contributed, though 67.5% of community variation remained unexplained. Because climate was strongly confounded with month and geography, this fraction should not be interpreted as evidence of causal dominance. The assembly of ladybird communities was associated with the interaction of climate and habitat, but the relative importance of climate versus habitat requires further experimental validation. These results provide a correlational foundation for understanding ladybird community assembly in oasis agriculture, but further experimental work is needed before they can be translated into specific management actions.

## Simple Summary

Ladybirds aid pest management on agricultural land. In the arid desert regions of southern Xinjiang, China, we examined the habitats of these ladybirds—croplands, orchards, shelterbelts, and weedy margin—and the effects of weather and seasonal changes on them. Over three years, we gathered more than 5400 ladybirds from 654 sites. Our research revealed that most were from two species, *Hippodamia variegata* and *Stethorus punctillum*, with populations peaking in June, significantly earlier than in cooler areas. Weather conditions, particularly cloud cover and wet day frequency, were significantly associated with ladybird presence and timing, and climate accounted for the largest unique fraction among the measured predictors, while habitat also contributed. These correlational patterns may help identify critical periods for conserving these pest controllers, although experimental validation is needed before specific management recommendations can be made.

## 1. Introduction

A primary challenge in macroecology is elucidating the patterns and mechanisms that sustain biodiversity, which is essential for developing effective conservation strategies [1,2,3]. This challenge is particularly pronounced in global drylands, where increasing climatic aridification causes rapid changes in ecosystem functioning, underscoring the extreme vulnerability of these regions to ongoing environmental changes [4,5,6,7]. Predatory natural enemy groups serve as highly informative indicators in examining the mechanisms that support biodiversity in arid ecosystems. As organisms at the upper trophic levels, they are more sensitive to environmental stress than herbivores, making their community assembly rules a valuable proxy for assessing the health of ecosystems.

Ladybirds (Coleoptera: Coccinellidae) are a significant group of arthropod predators that have been extensively studied because of their crucial role in controlling major agricultural pests, such as aphids and scale insects [8,9,10]. Previous studies have shown that ladybird community composition is influenced by local factors, including prey availability, climatic conditions, and the structural complexity of host plants [11,12]. However, a recognized limitation is that arable land is a highly disturbed and transient habitat, incapable of sustaining high levels of ladybird species diversity over extended periods [13]. This suggests that a comprehensive understanding of ladybird community structure necessitates research beyond croplands to include surrounding non-crop habitats. These habitats function as essential “reservoirs” for natural enemy populations by providing shelter, alternative prey, and overwintering sites, and are thus crucial for maintaining biodiversity in agricultural landscapes [14,15]. Indeed, habitat heterogeneity has been firmly established as a key predictor of biodiversity in human-managed landscapes [16].

In recent years, Chinese researchers have extensively surveyed ladybird assemblages within farmland ecosystems. Aerziguli·Rouzi et al. [17] conducted a two-year net-sweeping survey across 15 crop habitats in 50 counties across 11 prefectures in Xinjiang, collecting 7001 ladybird specimens from three subfamilies, 13 genera, and 26 species. *Hippodamia variegata* emerged as the dominant species, comprising 55.11% of the total individuals. Liangle Tang et al. [18] examined vegetable-growing habitats in northern Hainan, collecting 4993 ladybird individuals from three crop types, representing 16 genera and 28 species. Shulan Xu et al. [19] reported that certain flowering shrubs significantly attract adult natural enemies, including ladybirds, for supplementary feeding, suggesting a novel strategy for enhancing natural enemy populations through targeted vegetation management. In the Alar region of Xinjiang, Hujun Liang et al. [20] observed that dominant natural enemies, such as *Hippodamia variegata*, lacewings, and hoverflies, exhibited high temporal resource overlap with the mirid bug *Apolygus lucorum*, indicating a distinct prey-tracking pattern. Collectively, these studies have highlighted the significant influence of crop type and geographic region on ladybird communities. However, most surveys have been limited to single cropping systems or specific seasons, and comprehensive landscape-scale investigations integrating both spatial and temporal dynamics remain scarce.

The relationship between landscape complexity and biological control efficacy is more complex than previously assumed. Different natural enemy taxa exhibit substantial interspecific variation in their responses to landscape contexts [21,22], and increased natural enemy abundance or diversity does not consistently lead to enhanced pest suppression [23,24]. This uncertainty indicates that habitat heterogeneity alone is insufficient to fully explain the spatiotemporal variation in predator communities and that there may be underappreciated interactive effects between climatic factors, particularly seasonal drought stress, and land-use practices [25]. Ding Luo et al. [26] systematically reviewed how drought stress indirectly influences natural enemy population dynamics and biological control function by altering host plant physical traits, nutritional status, and secondary metabolites. Although these two lines of research approach the issue from different perspectives—physiological/mechanistic versus landscape-ecological—both have focused primarily on single factors, such as climate or landscape structure, without jointly considering how seasonal climate and habitat interact to shape predator communities. Wang et al. [27] demonstrated that in arid regions, changes in landscape patterns significantly affect the species composition and distribution of ground-dwelling beetle assemblages. However, most of these studies have adopted a univariate perspective, and direct field evidence is still lacking regarding how seasonal climatic fluctuations and habitat structure interactively drive the spatiotemporal dynamics of predator communities in extremely arid conditions.

The research gap is particularly evident in the ecologically sensitive oasis–desert transition zone of southern Xinjiang. This area has been persistently affected by the dual environmental challenges of desertification and salinization [28], leading to the establishment of extensive shelterbelt systems as essential ecological barriers [29]. Beyond their primary protective role, these artificial non-crop habitats are theoretically significant for conserving biological resources and enhancing regional biodiversity [30,31]. However, under an extreme continental climate, it remains uncertain whether the “reservoir” function of these artificial landscapes exhibits seasonal variation and how such variation interacts with periodic climatic fluctuations to influence the colonization and dispersal of ladybirds and other predatory arthropods in the region. Previous research has primarily focused on temperate agricultural systems, resulting in a significant lag in arthropod community studies in the Central Asian arid zone compared to regional ecological conservation needs.

To address these knowledge gaps, we conducted a systematic survey of ladybird assemblages in the Taklamakan Desert–oasis ecotone. Our central hypothesis posits that seasonal climatic fluctuations and habitat type exert significant interactive effects, collectively determining the spatiotemporal assembly patterns of ladybird communities. The specific objectives were as follows: (1) to characterize the species composition and alpha diversity of ladybirds in representative habitat types; (2) to quantify the spatiotemporal dynamics of ladybird assemblages across spatial (habitat type) and temporal (seasonal) scales; and (3) to disentangle the relative contributions of climatic factors and habitat structural variables, as well as their interactive effects, in shaping ladybird distribution patterns. The findings of this study are anticipated to provide crucial empirical evidence for advancing community assembly theory in dryland ecosystems and to offer scientific guidance for optimizing landscape management strategies aimed at enhancing biological control services. In contrast to previous surveys that were largely restricted to single cropping systems, single seasons, or temperate regions, the present study integrates a landscape-scale, multi-year dataset across four habitat types in the hyper-arid Taklamakan Desert–oasis ecotone. This design allows us to simultaneously quantify the relative contributions of habitat type, seasonal climate, and their shared variation to ladybird community assembly in an understudied arid agroecosystem.

## 2. Materials and Methods

### 2.1. Study Area and Site Design

Field sampling was conducted in oasis-agricultural ecosystems adjacent to the Taklamakan Desert in southern Xinjiang, China. The survey covered 29 counties and cities across five prefectures: Bayingolin Mongol Autonomous Prefecture, Aksu Prefecture, Kizilsu Kirghiz Autonomous Prefecture, Kashgar Prefecture, and Hotan Prefecture, spanning approximately 73–93° E and 35–42° N. Based on habitat characteristics, four site types were delineated: cropland, orchard, shelterbelt, and weedy margin. Cropland and orchard were classified as artificial habitats, whereas shelterbelt and weedy margin were considered semi-natural habitats.

Sampling was carried out from May to September in 2018, 2019, and 2024. Random sampling across the study region in 2018, 2019, and part of 2024 yielded a total of 654 sampling points (Table 1 and Table 2). Of these, 357 permanent sites were repeatedly sampled in 2024, 88 additional sites were surveyed once in 2024, and 209 sites were surveyed once in 2018–2019. The 357 permanent sites therefore constituted a subset of the 654 sampling points. In 2024, monthly surveys were additionally conducted at these permanent sites from June to September.

To examine seasonal variations in ladybird community diversity, three core monitoring counties were selected for monthly surveys from June to September 2024: Xinhe County (northern margin of the Tarim Basin), Yutian County (southern margin), and Shache County (southwestern margin). Within these counties, Systematic monthly surveys were conducted across all four habitat types at the 357 permanent sites. This subset captured the latitudinal and climatic gradients across the basin, thereby facilitating monthly analyses of community variation. To ensure spatial independence, any two plots within the same county were separated by a minimum distance of 2 km.

### 2.2. Specimen Collection and Identification

Ladybird specimens were collected using standard sweep nets, characterized by a 42 cm opening diameter and a 175 cm handle length. Surveys were conducted monthly between 10:00 and 13:00 local time. For tall crops such as maize, upward or oblique net sweeps were employed, with each sub-plot receiving 50 sweeps. In contrast, for short-to-medium-height vegetation, the net was positioned at a 30–60° angle relative to the ground and swept horizontally along a 180° arc, also with 50 sweeps per sub-plot. Consequently, each sampling point underwent a total of 150 net sweeps. Collected ladybirds were preserved in 95% ethanol, and geographic coordinates along with elevation were documented for each site. In the laboratory, specimens were mounted and pinned for subsequent taxonomic identification.

Species identification involved both morphological examination and molecular barcoding. Adult external morphological traits were examined using a Nikon SMZ 745T stereomicroscope (Nikon Corporation, Tokyo, Japan). The genitalia were dissected, treated in a 10% NaOH solution at 70 °C for approximately 30 min, and slide-mounted for observation. For specimens exhibiting ambiguous morphological traits or suspected cryptic species, the COI gene was amplified using two primer sets: LCO1490/HCO2198 and the ladybird-specific WDF/XSR [32]. The amplified products were sequenced by Beijing Tsingke Biotech Co., Ltd., Beijing, China.

### 2.3. Statistical Analyses

**Figure 1:**
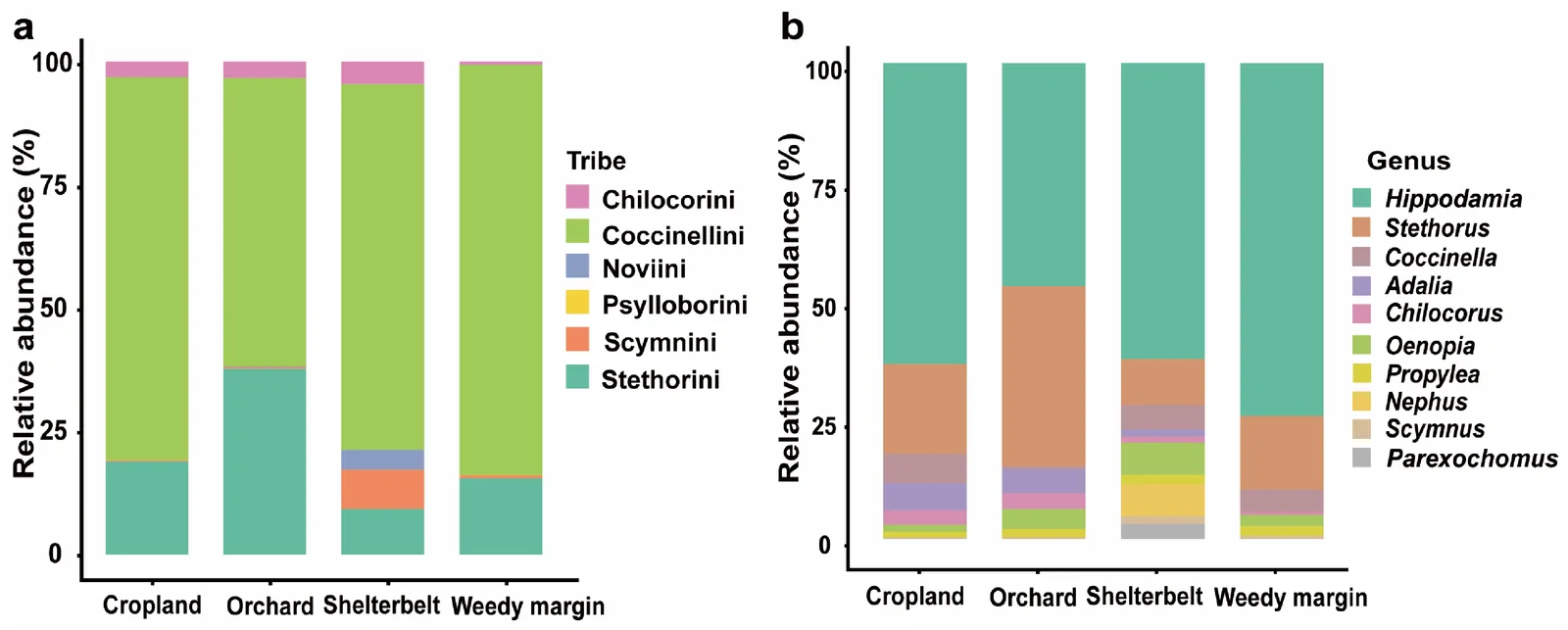
Relative abundance of ladybird communities in different habitats at the tribe (**a**) and genus (**b**) levels. Panel (**a**) shows tribe-level composition (Stethorini, Scymnini, Noviini, Chilocorini, Coccinellini, Psylloborini). Panel (**b**) shows genus-level composition.

**Figure 2:**
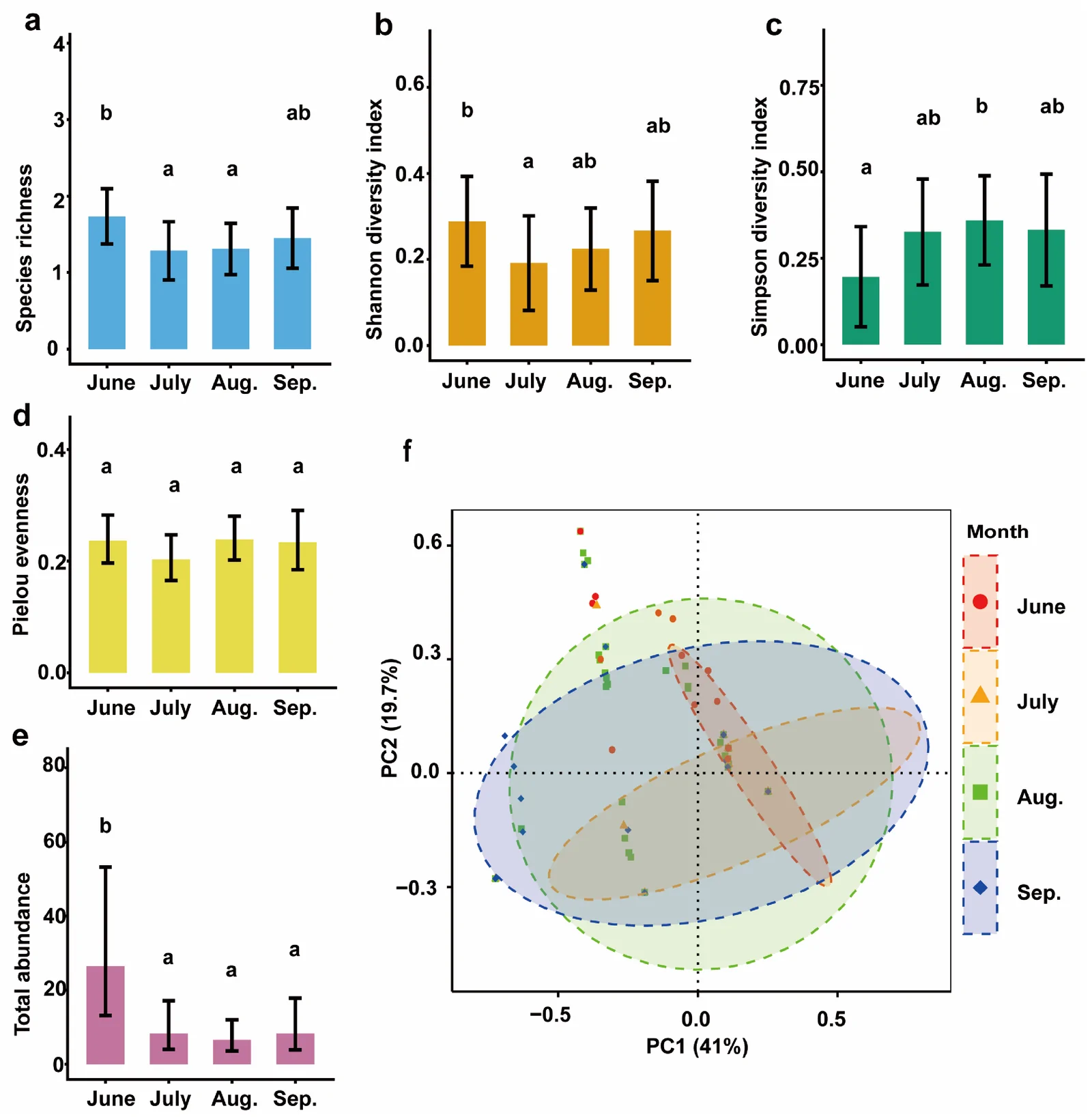
Monthly variations in α-diversity indices and β-diversity (PCoA) of ladybird communities. Panel (**a**) Species richness; (**b**) Shannon–Wiener index; (**c**) Simpson index; (**d**) Pielou evenness; (**e**) Total abundance; (**f**) PCoA ordination based on the Jaccard distance. Different lowercase letters indicate significant differences among months based on mixed-effects models followed by post-hoc comparisons (*p* < 0.05).

**Figure 3:**
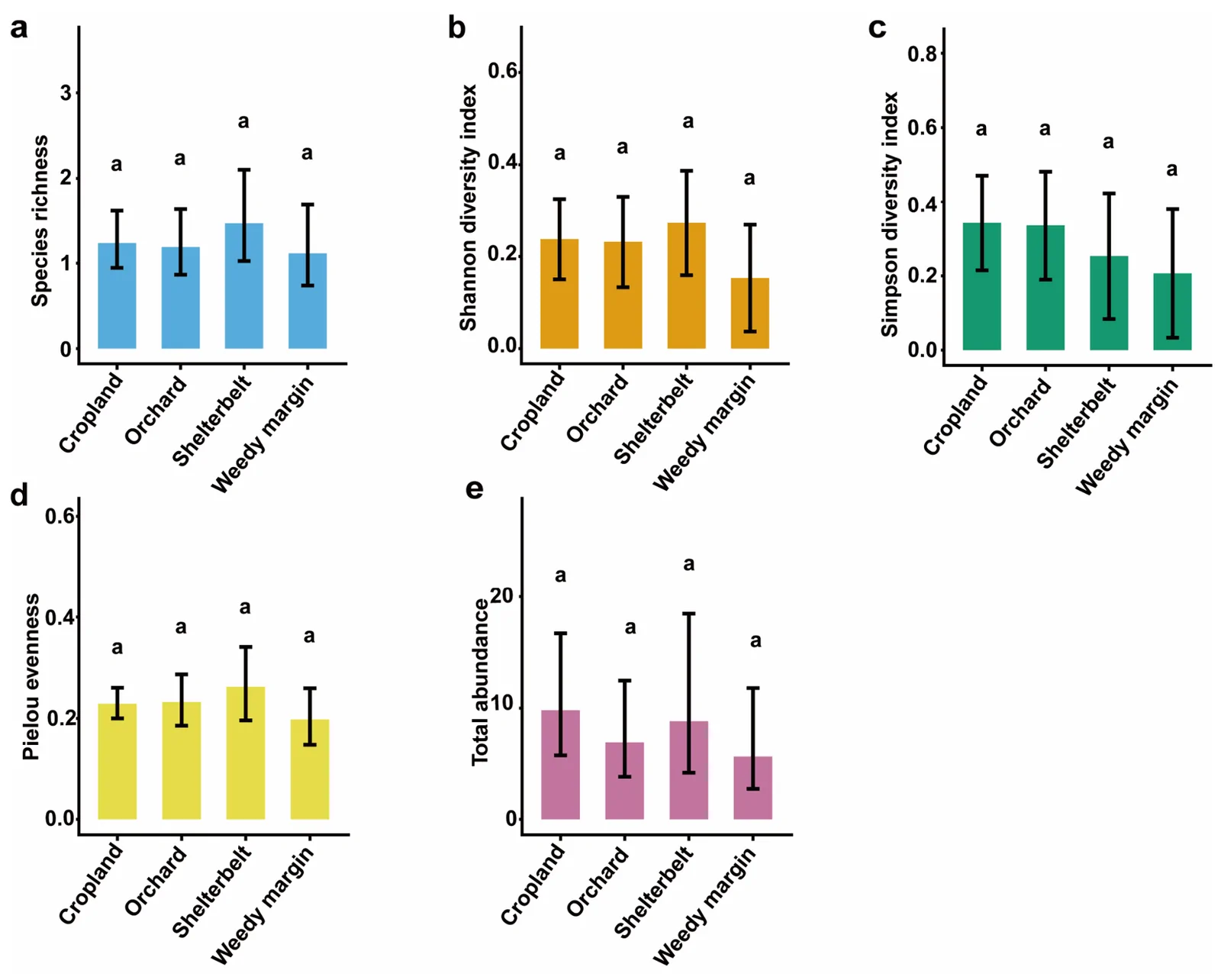
Presents a comparison of alpha diversity indices for ladybirds across various habitat types. Panel (**a**) Species richness; (**b**) Shannon–Wiener index; (**c**) Simpson index; (**d**) Pielou evenness; (**e**) Total abundance. Different lowercase letters indicate significant differences based on mixed-effects models (*p* < 0.05).

**Figure 4:**
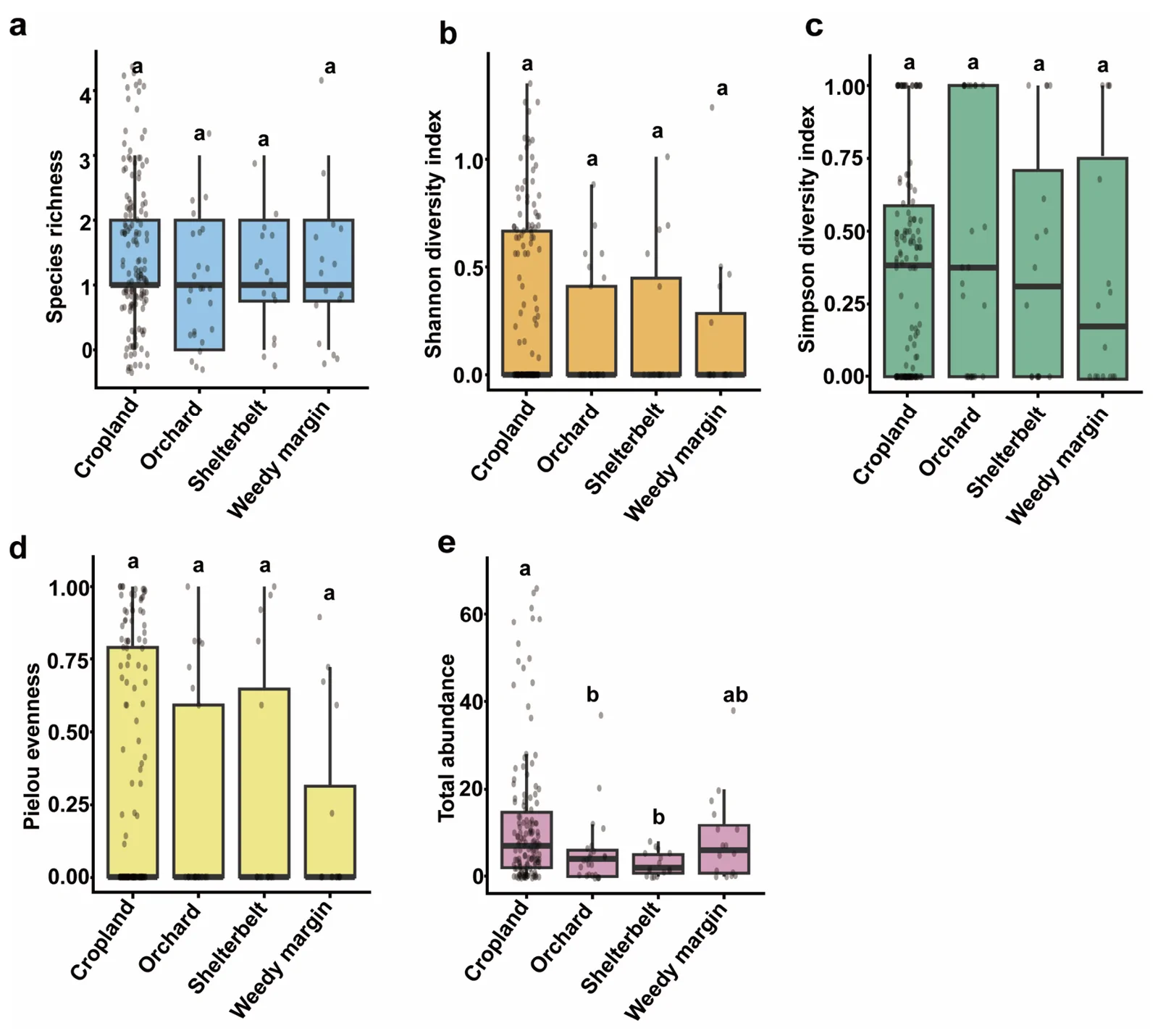
Presents a comparison of ladybird diversity indices across various habitats during the peak period in June. Panel (**a**) Species richness; (**b**) Shannon–Wiener index; (**c**) Simpson index; (**d**) Pielou evenness; (**e**) Total abundance. Different lowercase letters indicate significant differences based on Kruskal–Wallis tests followed by Dunn’s post-hoc tests with Benjamini–Hochberg FDR correction (*p* < 0.05).

**Figure 5:**
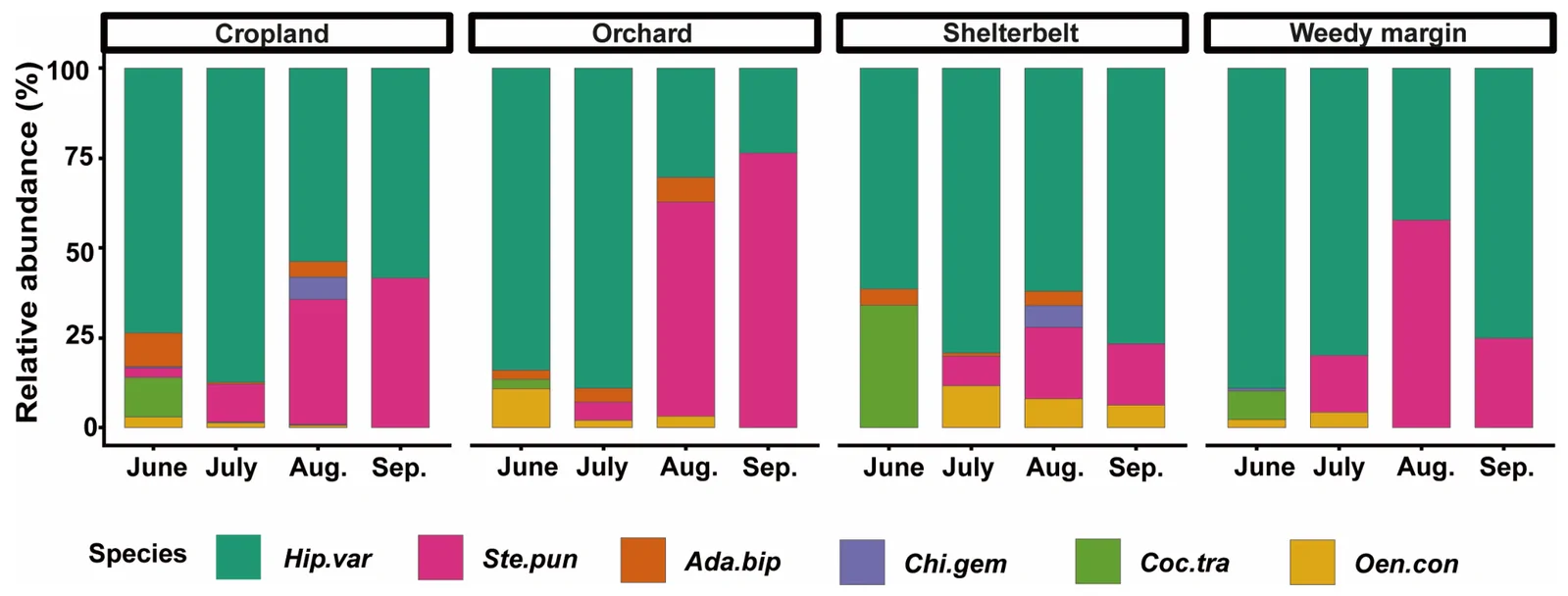
Changes in the relative abundance of dominant species across different habitats and months. Different colors represent different species. Species names are abbreviated as the first three letters of the genus and specific epithet, separated by a period. (e.g., *Hip.var* for *Hippodamia variegata*).

**Figure 6:**
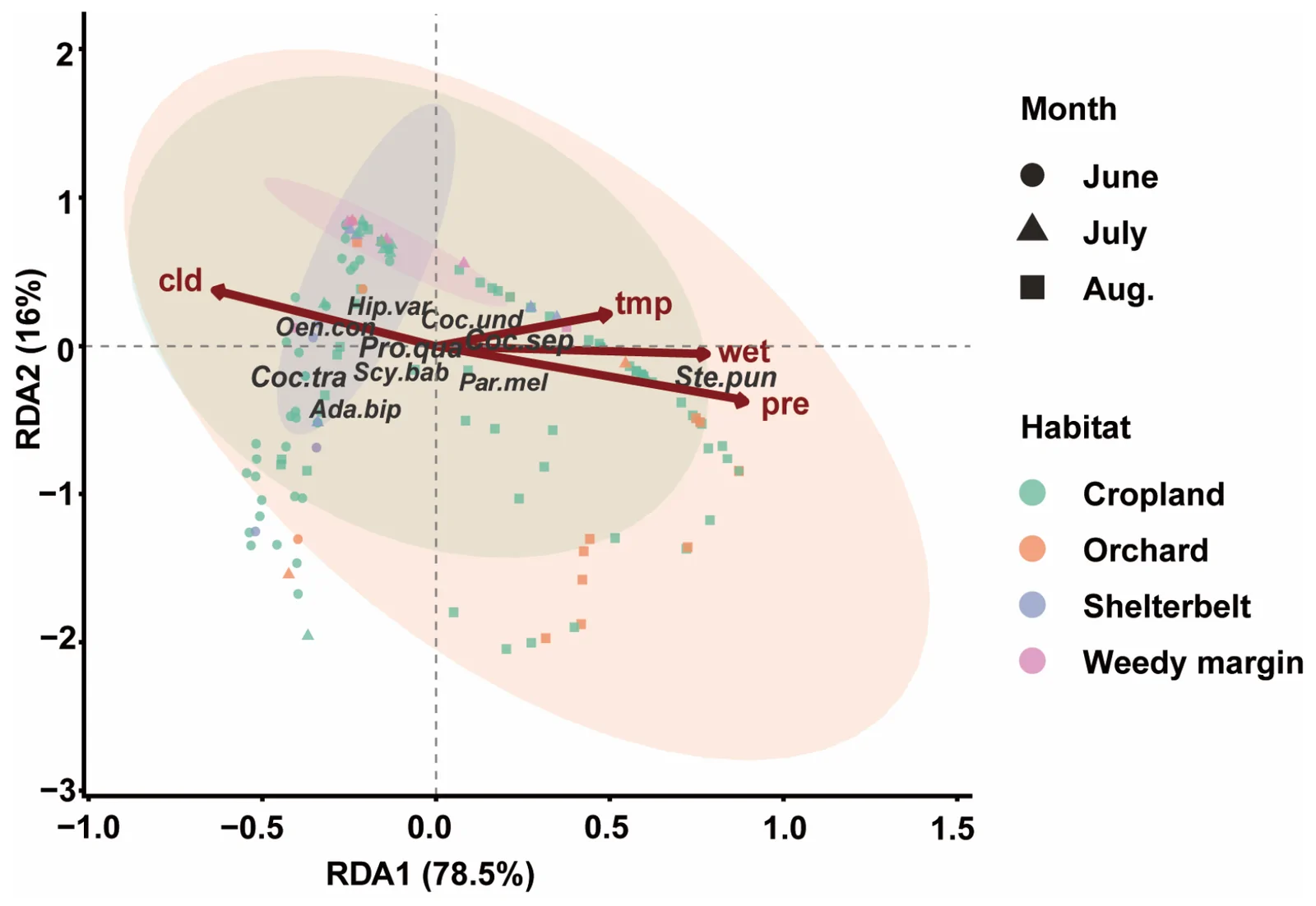
RDA ordination diagram of ladybird community composition and climatic factors. Axis 1 = RDA1 (78.5% of constrained variance); Axis 2 = RDA2 (16.0% of constrained variance). pre = precipitation (mm/month); cld = cloud cover (%); tmp = monthly average daily mean temperature (°C); wet = wet day frequency (days). Species abbreviations in black consist of the first three letters of the genus name and the first three letters of the specific epithet, separated by a dot (e.g., *Hip.var* = *Hippodamia variegata*).

Data sets and analytical subsets. Although field sampling was initially conducted from May to September, May samples were excluded prior to statistical analysis owing to insufficient sample size. All monthly analyses presented here therefore used June–September data, with month treated as a four-level factor (df = 3). Different analyses drew on different subsets of the data: the full 654-site dataset (Figures 1 and 5, Tables 5 and 6); the 357 permanent sites sampled in 2024 (Figures 2 and 3); June samples from the 654-site dataset (Figure 4); The 2018–2019 dataset comprising 209 sites (Figure 6, Tables 7 and 8).

Species were classified into three ecological groups based on their relative abundance: dominant species (relative abundance >10%), common species (1–10%), and rare species (<1%). This classification follows the McNaughton dominance index approach commonly used in ladybird community studies, in which species with a dominance index >0.01 are considered dominant [33,34].

All statistical analyses were conducted using R (version 4.3.1) with the appropriate R packages for data visualization.

#### 2.3.1. Community Diversity Analysis

To characterize alpha diversity at each sampling site, we calculated species richness, the Shannon–Wiener index, the Simpson index, Pielou’s evenness, and total abundance. Differences in these diversity metrics across months and habitat types were evaluated using Kruskal–Wallis tests, followed by Dunn’s post-hoc tests. Multiple comparisons were adjusted using the Benjamini–Hochberg false-discovery-rate (FDR) procedure, with significance determined at α = 0.05. Beta diversity was assessed using Jaccard distance. Community compositional differences among months were analyzed using PERMANOVA with 999 permutations. Because the 357 permanent sites were repeatedly sampled across months, the repeated-measures structure of the data should be acknowledged when interpreting PERMANOVA results. PERMANOVA indicated a highly significant effect of month on community composition (*p* < 0.001). Principal coordinate analysis (PCoA) based on Jaccard distance was employed to visualize community compositional similarity.

#### 2.3.2. Mixed-Effects Models

Linear mixed-effects models were developed to examine the individual and interactive effects of habitat and month on ladybird diversity indices. Each diversity metric was used as the response variable, with habitat, month, and their two-way interaction included as fixed effects, and sampling-site ID specified as a random intercept. Models were fitted using the lme4 package. Total abundance and species richness are count variables and were modeled with negative binomial GLMMs to account for overdispersion. Shannon and Simpson diversity indices were modeled with Gaussian LMMs after checking residual normality and homoscedasticity. Pielou evenness is a bounded continuous variable and was modeled with a beta regression mixed model using the glmmTMB package (R version 4.4.2). Habitat, month, and their two-way interaction were included as fixed effects, and sampling-site ID was specified as a random intercept to account for repeated measures. Type II Wald χ^2^ tests, implemented via the car package, were used to generate model outputs.

Separate models with identical structures were constructed for the six most abundant dominant species to evaluate how their relative abundances responded to habitat and month.

#### 2.3.3. Species–Environment Relationships

Distance-based redundancy analysis (db-RDA) was performed to quantify the influence of climate and habitat on ladybird community structure. Climate predictors included cloud cover (cld), wet day frequency (wet), monthly average daily mean temperature (tmp), and precipitation (pre). Climate data were obtained from the Climatic Research Unit (CRU) TS v4.07 high-resolution gridded dataset, University of East Anglia [35] (https://crudata.uea.ac.uk/cru/data/hrg/cru_ts_4.07/cruts.2304141047.v4.07/ accessed on 24 November 2025). The dataset has a spatial resolution of 0.5° × 0.5°. Variables were recorded as follows: precipitation (pre, mm/month), monthly average daily mean temperature (tmp, °C), cloud cover (cld, %), and wet day frequency (wet, days). For each sampling event, climatic variables were extracted for the grid cell containing the sampling site and matched to the corresponding month of sampling. Because climate is strongly confounded with month and geography, we interpreted the climate fraction as the unique fraction explained among the measured predictor sets rather than as a causal dominant driver. PERMANOVA permutations were not constrained by site, and multivariate dispersion was not formally tested; therefore, the month effect should be interpreted with caution. Forward selection retained only significant explanatory variables (*p* < 0.05). The community matrix for db-RDA was constructed using species abundance data with Jaccard distance, utilizing functions from the vegan package. Variation-partitioning analysis (VPA) was further conducted with varpart (vegan) to decompose the variance explained by climate, habitat, month, and their joint effects. Because climate is strongly confounded with month and geography, the climate fraction was interpreted as the unique fraction explained among the measured predictor sets rather than as a causal dominant driver.

## 3. Results

### 3.1. Community Composition and Structural Characteristics

The composition of ladybird communities in southern Xinjiang is presented in Table 3. The survey collected 5414 ladybird specimens, encompassing 23 species, 14 genera, and seven tribes. Among these, Coccinellini was predominant, comprising 11 species (47.83% of total species) and 4109 individuals (75.90% of total abundance). Stethorini was the second most abundant tribe, with 1 species (4.35%) and 1085 individuals (20.04%). Scymnini included 5 species (21.74%) but only 38 individuals (0.70%), while Chilocorini comprised 4 species (17.39%) and 165 individuals (3.05%). Noviini and Psylloborini were the least common, each with 1 species (4.35%) and 14 or 3 individuals (0.26% or 0.06%), respectively (Table 3). *Hippodamia* exhibited the highest individual abundance, with 3265 individuals constituting 60.3% of the total specimens. This was followed by *Stethorus* (1085 individuals, 20.0%) and *Coccinella* (284 individuals, 5.2%), whereas other genera contributed minimally to the overall abundance.

Analysis of species abundance indicated that *Hippodamia variegata* was the dominant species, comprising 60.31% of all collected individuals. The subdominant species included *Stethorus punctillum* (20.04%), *Adalia bipunctata* (4.95%), *Coccinella transversoguttata* (3.90%), *Chilocorus geminus* (2.33%), and *Oenopia conglobata* (2.16%). Together, these six species accounted for 93.68% of the total specimens, whereas the remaining 17 species contributed only 6.32% of the total abundance (Table 4). Based on relative abundance, ladybirds were classified into three ecological groups: *H. variegata* and *S. punctillum* were identified as dominant species; *A. bipunctata*, *C. transversoguttata*, *C. geminus*, *O. conglobata*, *Adalia decempunctata*, *Propylea quatuordecimpunctata*, and *Coccinella undecimpunctata* were categorized as common species; the remaining 17 species (e.g., *Chilocorus renipustulatus*, *Nephus incinctus*) with very low abundance were considered rare species.

The community structure of ladybirds at the tribe and genus levels exhibited distinct variations across different habitat types (Figure 1). In terms of tribe composition, Coccinellini maintained a relative abundance exceeding 70% in cropland, shelterbelt, and weedy margin habitats, reaching its highest value in weedy margins (approximately 80%). However, its dominance decreased markedly in orchard habitats, where the relative abundance was approximately 65%, while Stethorini increased to approximately 35% in orchard habitats (Figure 1a). At the genus level, *Hippodamia* was consistently dominant across all habitat types, with its relative abundance surpassing 60% in croplands and orchards.

The genus *Stethorus* displayed a clear habitat preference, achieving higher abundance in shelterbelts (28%) and weedy margins (25%). The genus *Coccinella* accounted for 5–8% of total individuals in cropland and orchard habitats, while all other genera maintained consistently low relative abundance across all surveyed habitats (Figure 1b).

### 3.2. Spatiotemporal Dynamics of Ladybird Diversity

Ladybird diversity indices showed pronounced monthly fluctuations (Figure 2a–e). Species richness differed significantly among months (χ^2^ = 15.780, df = 3, *p* = 0.001). Post-hoc comparisons indicated that June (1.75) was significantly higher than in July (1.03) and August (1.15) (*p* < 0.05), but not significantly different from September (1.30). The monthly difference in total abundance was most pronounced: June (26.47) was significantly higher than July–September (means 7.5–8.4, χ^2^ = 41.747, df = 3, *p* < 0.001), with no significant differences among July–September. The Shannon index showed a marginal month effect (χ^2^ = 7.473, df = 3, *p* > 0.05); post-hoc comparisons indicated that June (0.289) was significantly higher than July (0.191, *p* < 0.05), but not significantly different from August (0.224) or September (0.266). The Simpson index differed significantly among months (χ^2^ = 12.283, df = 3, *p* < 0.01); post-hoc comparisons indicated that June (0.196) was significantly lower than August (0.360, *p* < 0.01), with no significant differences among other months. Pielou evenness did not differ significantly among months (χ^2^ = 2.038, df = 3, *p* > 0.05; all months shared the same grouping symbol). PCoA ordination based on Jaccard distance (Figure 2f) separated June samples from July–September samples; PERMANOVA indicated a significant month effect on community composition (*p* < 0.001), although permutations were not constrained by site and multivariate dispersion was not formally tested.

Comparisons among the four habitat types showed no significant differences for any diversity index (Kruskal–Wallis test, *p* > 0.05; Figure 3). To further disentangle the effects of habitat, month, and their interaction, mixed-effects models were applied (Table 5). Total abundance was significantly associated with month (χ^2^ = 41.747, df = 3, *p* < 0.001), while habitat and the habitat × month interaction were not significant (*p* > 0.05). The Simpson index was significantly associated only with month (χ^2^ =12.283, df = 3, *p* < 0.05), whereas habitat and the interaction were not significant. Species richness was significantly associated with month (χ^2^ = 15.780, df = 3, *p* < 0.001), but not with habitat or the interaction. The Shannon index showed a marginal month effect (χ^2^ = 7.473, df = 3, *p* > 0.05), and Pielou evenness showed no significant effects of habitat, month, or their interaction (*p* > 0.05).

Given that June represented the peak period of ladybird activity, data from June were analyzed separately (Figure 4). Among-habitat differences were significant only for total abundance (χ^2^ = 13.42, *p* < 0.01), with cropland supporting significantly higher abundance than orchards and shelterbelts (Dunn’s test, adjusted *p* < 0.05). No other diversity metrics differed across habitats in June.

### 3.3. Differential Responses of Dominant Species to Season and Habitat

We developed distinct linear mixed-effects models for the six most prevalent species (*H. variegata*, *A. bipunctata*, *C. geminus*, *S. punctillum*, *C. transversoguttata*, and *O. conglobata*) to evaluate how their relative abundances were influenced by habitat and month (Table 6). The month of observation had a highly significant impact on *H. variegata* (χ^2^ = 9.814, df = 3, *p* < 0.05), *S. punctillum* (χ^2^ = 40.725, df = 3, *p* < 0.001), and *C. transversoguttata* (χ^2^ = 9.930, df = 3, *p* < 0.001). For *C. geminus*, neither habitat nor month nor their interaction was significant (*p* > 0.05). For *O. conglobata*, habitat had a significant main effect (χ^2^ = 8.279, df = 3, *p* < 0.05), while month and the interaction were not significant. *A. bipunctata* showed no significant response to any factor.

Stacked bar charts illustrate the seasonal turnover of these six dominant species across habitats (Figure 5). Overall, *H. variegata* was predominant in croplands and orchards throughout the sampling season, although its relative dominance decreased over time. *S. punctillum* experienced a sharp increase in August, particularly within croplands (34.9%) and weedy margins (~20%), indicating strong seasonal growth. In orchards, *A. bipunctata* and *C. transversoguttata* increased their relative shares during July–August. The species composition in the shelterbelts was relatively balanced, with no single taxon achieving absolute dominance. These observations underscore the interspecific divergence in seasonal responses. Habitat type had a significant main effect only for *O. conglobata*, whereas month was the primary driver for *H. variegata*, *S. punctillum*, and *C. transversoguttata*.

### 3.4. Climatic Drivers of Ladybird Communities

Mixed-effects models were employed to evaluate the influence of climate and habitat on ladybird diversity indices (Table 7). Both cloud cover and wet day frequency had significant effects on species richness (*p* < 0.01). Wet day frequency was a significant predictor of both the Shannon–Wiener index (*p* < 0.05) and Pielou’s evenness (*p* < 0.05). Total abundance was influenced by cloud cover (*p* < 0.001), monthly average daily mean temperature (*p* < 0.05), and habitat (*p* < 0.001).

The global db-RDA model demonstrated statistical significance (F = 6.996, *p* < 0.001). For clarity, the first and second constrained axes are hereafter referred to as RDA1 and RDA2, respectively (Axis 1 = RDA1; Axis 2 = RDA2). RDA1 accounted for 78.5% of the constrained variance and RDA2 for 16.0% (Figure 6). Along RDA1, precipitation (pre) and wet day frequency (wet) showed the strongest positive associations, with monthly average daily mean temperature (tmp) also loading positively, whereas cloud cover (cld) loaded primarily in the opposite direction. *S. punctillum* was positioned toward the positive end of RDA1, whereas *H*. *variegata*, *O*. *conglobata*, *C*. *transversoguttata*, and *A*. *bipunctata* were positioned near the origin or toward the negative end of RDA1. Samples from cropland predominantly clustered at the positive end of RDA1.

Variation-partitioning results (Table 8) indicated that climate, habitat, and month collectively accounted for 32.5% of the variance in community composition. The climate-only fraction was the largest unique component among the measured predictor sets (14.2%), followed by the joint habitat–month component (13.0%). The three-way shared fraction accounted for 1.7%, while the climate–habitat joint effects explained 3.2%. Because climate was strongly confounded with month and geography, the climate-only fraction should be interpreted as the unique variance explained by the measured climatic predictor set rather than as evidence of a single causal climatic driver. These fractions represent shared explained variance, not statistical causation.

## 4. Discussion

This study provides a detailed examination of the spatiotemporal patterns and climatic influences on ladybird assemblages within the Taklamakan Desert–oasis ecotone, which is recognized as one of the most arid agricultural landscapes globally. *H*. *variegata* and *S*. *punctillum* constituted 80.35% of the collected specimens, indicating a higher species concentration compared to ladybird communities in eastern-China agroecosystems [18]. Extreme aridity imposes significant environmental filtering, allowing only ladybirds with high dispersal capabilities and broad physiological tolerance to maintain stable populations in this climatically volatile desert–oasis transition zone [5,7].

The type of habitat influences community composition. Coccinellini were predominant in cropland shelterbelts and weedy margin habitats, whereas Stethorini were more abundant in orchard habitats. During peak crop-growing periods, croplands and orchards support dense aphid populations, which promote the aggregation and reproduction of Coccinellini such as *H. variegata* [10]. In contrast, orchards, characterized by structurally complex vegetation and lower prey availability, support a higher proportion of small-bodied Stethorini (e.g., *Stethorus*) that are less dependent on dense prey populations [14]. Despite the anticipated greater compositional heterogeneity in semi-natural habitats [16], no significant differences in alpha-diversity indices were observed across the four habitat categories. This pattern may result from the overwhelming numerical dominance of a few species across all habitats, obscuring habitat-driven alpha-diversity differences. Habitat-related compositional shifts primarily affected the relative abundances of common and rare species [36], with *O. conglobata* showing a significant habitat main effect in the species-level models. Similar findings have been reported for ground beetle assemblages in arid regions [27].

The seasonal trajectories of ladybird communities in this arid system differed from those documented in temperate agroecosystems. All diversity indices peaked in June and subsequently declined, whereas temperate ladybird diversity typically reaches its maximum during July–August [10]. This earlier phenological peak aligns with recent observations of advanced biotic seasonality in dryland ecosystems [37]. June coincides with the peak growth of local crops in southern Xinjiang, when aphid prey are abundant [26], and thermal–hydric conditions remain favorable, preceding the intense heat and moisture stress of July–August [38]. The decline in diversity post-June may be attributed to habitat disturbance associated with crop harvest, prey depletion, and adult mortality or dispersal. Mixed-model outputs further indicate that seasonal changes were primarily driven by shifts in the relative abundance of *H. variegata*, *S. punctillum*, and *C. transversoguttata*, rather than a complete turnover of species.

Variation-partitioning results indicated that climate accounted for more unique variance than either habitat or month, although the joint effects of habitat and month were also significant. The intense summer heat and low wet day frequency characteristics of this desert–oasis ecotone impose strong physiological constraints, allowing only heat- and drought-tolerant species to persist locally [4]. In the mixed-effects models, cloud cover and wet day frequency were significant predictors of species richness, whereas total abundance was influenced by cloud cover, monthly average daily mean temperature, and habitats. Additionally, wet day frequency was a predictor of Shannon–Wiener diversity and Pielou’s evenness. The non-significant direct effect of monthly average daily mean temperature on species richness, together with the significant effects of cloud cover and wet day frequency, suggests that water availability rather than monthly average daily mean temperature per se may be the primary limiting factor for ladybird activity in this hyper-arid environment. This interpretation is supported by previous findings from Xinjiang, where water loss, rather than ambient monthly average daily mean temperature, was associated with population decline in local ladybirds [39]. Specifically, *H. variegata* (the dominant species in our study) loses water at 1.7–2.3 times the rate of *C. septempunctata* under identical monthly average daily mean temperature treatments, and field investigations showed that water from food, rather than ambient monthly average daily mean temperature, was the likely cause of ladybird population crashes. In such environments, cloud cover reduces solar radiation and evaporative water loss, while more frequent wet days may partially compensate for cuticular water loss, thereby extending the window of ladybird activity. Monthly average daily mean temperature may therefore act indirectly through its effects on prey populations rather than directly on ladybird abundance. These correlational findings underscore water availability as an important measured factor structuring arid-zone ladybird assemblages [40]. A substantial portion of community variance (67.5%) remained unexplained by our predictors, indicating that unmeasured variables—such as prey density, interspecific interactions, and stochastic dispersal—may contribute importantly to assemblage organization [41].

Dominant species exhibited varied seasonal–habitat responses. Season had a significant effect on *H. variegata*, *S. punctillum*, and *C. transversoguttata*, but not on *C. geminus*, *A. bipunctata*, or *O. conglobata*. *H. variegata* was prevalent in cropland and orchard but decreased in relative abundance as summer advanced, whereas *S. punctillum* increased markedly in late summer. This opposing seasonal trend may be linked to its specialization on spider-mite prey, which often proliferates under hot–dry late-summer conditions [42]. In contrast to our initial expectation, *C. geminus* did not show a significant habitat–month interaction in the revised models, suggesting that its seasonal dynamics may be more uniform across habitats than previously thought. *O. conglobata*, by contrast, showed a significant habitat main effect, indicating that its relative abundance varies more strongly among habitat types than across months. The lack of significant responses for *A. bipunctata* is consistent with generalist feeding strategies [43]. Such differentiation in responses among dominant taxa may contribute to the stabilization of ladybird assemblages within arid agroecosystems.

Our findings provide correlational insights that may inform natural-enemy conservation and landscape planning within southern Xinjiang oasis farmland. Because ladybird abundance and diversity peak in June, this period may represent a critical window for conserving resident predator populations; however, experimental validation is needed before translating these patterns into specific management recommendations. Similarly, the late-summer increase in *S. punctillum* suggests a potentially important period for conservation, but further work is required to confirm whether this pattern can be leveraged for pest suppression.

Several limitations should be acknowledged. First, PERMANOVA permutations were not constrained by site, and multivariate dispersion was not formally tested; therefore, the month effect on community composition should be interpreted with caution. Second, climatic variables were derived from a monthly gridded dataset (CRU TS) and matched to the corresponding month of sampling rather than to the exact sampling date, which may introduce temporal mismatch between climate exposure and ladybird sampling. Third, a substantial portion of community variation (67.5%) remained unexplained, indicating that unmeasured factors such as prey density, interspecific interactions, and stochastic dispersal may play important roles. Finally, our management suggestions are correlational and require experimental validation before being applied in practice.

## 5. Conclusions

In conclusion, seasonal climate fluctuations and habitat type together explained a significant but moderate portion of the spatiotemporal variation in ladybird assemblages across the Taklamakan Desert–oasis ecotone. Climate explained the largest unique fraction among the measured predictor sets, while 67.5% of the variation remained unexplained. The habitat–month and three-way fractions represented shared explained variation rather than direct statistical interactions. The differing seasonal and habitat responses among the dominant species indicate species-specific phenological and habitat associations; however, niche partitioning was not directly tested in this study, which provides empirical evidence of ladybird assembly in hyper-arid ecosystems and offers scientific support for biocontrol resource management and landscape optimization within Xinjiang oasis agricultural systems.

## Figures and Tables

**Table 1:** Distribution of sampling sites in various regions of southern Xinjiang.

Prefecture	Counties/Cities Covered	Longitude Range	Latitude Range	No. of Sites
Bayingolin	Korla, Luntai, Yuli, Qiemo, Tiemenguan, Nantun, Hoxud, Yanqi, Bohu	85–93° E	38–42° N	70
Aksu	Aksu, Wensu, Kuqa, Xinhe, Alar	79–83° E	40–42° N	192
Kizilsu	Artux	73–78° E	39–41° N	5
Kashgar	Kashgar, Shule, Yengisar, Zepu, Shache, Yecheng, Yuepuhu	73–79° E	37–40° N	229
Hotan	Hotan City, Hotan County, Karakax, Pishan, Lop, Yutian, Minfeng	79–83° E	35–39° N	158
Total	29 counties/cities	—	—	654

**Table 2:** Habitat types and number of sampling sites in the study area.

Habitat Type	Specific Description	Category	No. of Sites	Habitat Type
Cropland	Wheat, maize, cotton, alfalfa	Artificial	422	Cropland
Orchard	Walnuts, jujubes, apples, apricots, peaches	Artificial	105	Orchard
Shelterbelt	Farmland shelterbelts (Populus, Ulmus)	Semi-natural	65	Shelterbelt
Weedy margin	Natural or semi-natural weedy vegetation along field edges	Semi-natural	62	Weedy margin
Total	—	—	654	Total

**Table 3:** Composition of ladybird communities at the tribe level in southern Xinjiang.

Tribe	No. of Genera	Proportion (%)	No. of Species	Proportion (%)	No. of Individuals	Proportion (%)
Stethorini	1	7.14	1	4.35	1085	20.04
Scymnini	2	14.29	5	21.74	38	0.70
Noviini	1	7.14	1	4.35	14	0.26
Chilocorini	3	21.43	4	17.39	165	3.05
Coccinellini	6	42.86	11	47.83	4109	75.90
Psylloborini	1	7.14	1	4.35	3	0.06
Total	14	100	23	100	5414	100

**Table 4:** Species list and individual numbers of ladybird communities in southern Xinjiang.

Tribe	Genus	Species	No. of Individuals	Proportion (%)
Stethorini	*Stethorus*	*S. punctillum*	1085	20.04
Scymnini	*Scymnus*	*S. mongolicus*	8	0.15
		*Scymnus* sp.	1	0.02
		*S. frontalis*	1	0.02
		*S. babai*	7	0.13
	*Nephus*	*N. incinctus*	21	0.39
Noviini	*Novius*	*N. limbatus*	14	0.26
Chilocorini	*Chilocorus*	*C. renipustulatus*	24	0.44
		*C. geminus*	126	2.33
	*Parvexochomus*	*P. semenovi*	14	0.26
	*Brumus*	*B. octosignatus*	1	0.02
Coccinellini	*Hippodamia*	*H. tredecimpunctata*	4	0.07
		*H. variegata*	3265	60.31
		*H. arctica*	1	0.02
	*Adalia*	*A. decempunctata*	99	1.83
		*A. bipunctata*	268	4.95
	*Propylea*	*P. quatuordecimpunctata*	70	1.29
	*Oenopia*	*O. conglobata*	117	2.16
	*Coccinella*	*C. septempunctata*	5	0.09
		*C. undecimpunctata*	68	1.26
		*C. transversoguttata*	211	3.90
	*Harmonia*	*H. axyridis*	1	0.02
Psylloborini	*Psyllobora*	*vigintiduopunctata*	3	0.06

**Table 5:** Presents the results of mixed-effects models for the diversity indices of ladybird communities, analyzed in relation to habitat and month using Type II Wald chi-square tests.

Index	Effect	χ^2^	df	*p*-Value
Species richness	Habitat	2.352	3	0.503
	Month	15.780	3	0.001
	Habitat × Month	11.898	9	0.220
Shannon index	Habitat	4.031	3	0.258
	Month	7.473	3	0.058
	Habitat × Month	11.093	9	0.269
Simpson index	Habitat	7.047	3	0.070
	Month	12.283	3	0.006
	Habitat × Month	13.767	9	0.131
Pielou evenness	Habitat	2.096	3	0.553
	Month	2.038	3	0.565
	Habitat × Month	5.198	9	0.817
Total abundance	Habitat	4.090	3	0.252
	Month	41.747	3	<0.001
	Habitat × Month	15.074	9	0.089

**Table 6:** Results of linear mixed-effects models for relative abundance of dominant species in relation to habitat and month (Type II Wald chi-square tests).

Index	Effect	χ^2^	df	*p*-Value
*Hippodamia variegata*	Habitat	**1.** **421**	3	**0.** **701**
	Month	**9.814**	3	**0.** **020**
	Habitat × Month	**10.219**	9	**0.** **333**
*Adalia bipunctata*	Habitat	**6.** **443**	3	**0.** **092**
	Month	**0.936**	3	**0.** **817**
	Habitat × Month	**9.199**	9	**0.** **419**
*Chilocorus geminus*	Habitat	**4.111**	3	**0.** **250**
	Month	**3.432**	3	**0.** **327**
	Habitat × Month	**5.124**	9	**0.** **823**
*Stethorus punctillum*	Habitat	**3.402**	3	**0.** **334**
	Month	**40.725**	3	**<0.001**
	Habitat × Month	**10.983**	9	**0.** **280**
*Coccinella transversoguttata*	Habitat	**2.497**	3	**0.** **476**
	Month	**9.930**	3	**0.** **019**
	Habitat × Month	**4.665**	9	**0.** **862**
*Oenopia conglobata*	Habitat	**8.279**	3	**0.** **041**
	Month	**4.770**	3	**0.** **189**
	Habitat × Month	**7.619**	9	**0.** **573**

**Table 7:** Results of mixed-effects models for climatic factors and habitat on ladybird community diversity indices (Type II Wald chi-square tests).

Index	Effect	χ^2^	df	*p*-Value
Species richness	pre	0.009	1	0.922
	cld	8.22	1	0.004
	tmp	0.206	1	0.650
	wet	8.312	1	0.004
	habitat	0.118	3	0.990
Shannon index	pre	0.028	1	0.866
	cld	3.812	1	0.051
	tmp	0.035	1	0.851
	wet	6.57	1	0.010
	habitat	3.312	3	0.346
Simpson index	pre	0.563	1	0.453
	cld	0.368	1	0.544
	tmp	0.005	1	0.944
	wet	1.856	1	0.173
	habitat	2.724	3	0.436
Pielou evenness	pre	0.632	1	0.427
	cld	0.384	1	0.535
	tmp	0.241	1	0.623
	wet	5.141	1	0.023
	habitat	5.622	3	0.131
Total abundance	pre	0.061	1	0.804
	cld	23.912	1	<0.001
	tmp	5.319	1	0.021
	wet	0.69	1	0.406
	habitat	22.217	3	<0.001

Note: pre = precipitation (mm/month); cld = cloud cover (%); tmp = monthly average daily mean temperature (°C); wet = wet day frequency (days).

**Table 8:** Results of variation partitioning for climatic factors, habitat, and month on ladybird community composition (adjusted R^2^).

Index	Effect	Adjusted R^2^
Climate alone	0.142	14.2
Habitat alone	0.004	0.4
Month alone	0	0.0
Climate ∩ Habitat	0.032	3.2
Climate ∩ Month	0.0	0.0
Habitat ∩ Month	0.130	13
Combined (Climate ∩ Habitat ∩ Month)	0.017	1.7
Total explained	0.325	32.5
Unexplained	0.675	67.5
Total	1	100

Note: Fractions represent shared explained variance, not statistical causation. The climate-only fraction is the unique fraction explained among the measured predictor sets.

## Data Availability

The original contributions presented in this study are included in the article. Further inquiries can be directed to the corresponding authors.
